# Design and Preparation of Inherently Photostable Poly(Butylene Adipate-Co-Terephthalate) by Chemically Bonding UV-Stabilizing Moieties in Molecular Chains

**DOI:** 10.3390/polym17111567

**Published:** 2025-06-04

**Authors:** Xinpeng Zhang, Yan Ye, Yaqiao Wang, Hongli Bian, Jing Yuan, Jianping Ding, Wanli Li, Jun Xu, Baohua Guo

**Affiliations:** 1 Key Laboratory of Advanced Materials (MOE), Department of Chemical Engineering, Tsinghua University, Beijing 100084, China; zhangxp20@mails.tsinghua.edu.cn (X.Z.); yeyan23@mails.tsinghua.edu.cn (Y.Y.); w646277458@126.com (Y.W.); hongli-bian@mail.tsinghua.edu.cn (H.B.); yuanjing0780@163.com (J.Y.); 2 Xinjiang Blue Ridge Tunhe Sci. & Tech. Co., Ltd., Changji 831199, China; 123303043@163.com (J.D.); liwanli0204@163.com (W.L.)

**Keywords:** PBAT, covalent linking, biodegradable, UV resistance, inherent property

## Abstract

Poly(butylene adipate-co-terephthalate) (PBAT) is a promising biodegradable polymer with balanced mechanical properties and excellent degradability, making it an ideal material to reduce plastic pollution. However, its susceptibility to ultraviolet (UV) degradation, due to photosensitive aromatic rings and carbonyl groups in its structure, limits its use in outdoor settings like mulch films. Conventional methods of incorporating small-molecule UV stabilizers face challenges such as poor compatibility, uneven dispersion, and migration under environmental conditions, reducing their effectiveness over time. This study developed a novel strategy to enhance PBAT’s UV resistance by chemically bonding UV-stabilizing moieties directly into its molecular chains to address these limitations. A novel UV absorber containing a polymerizable group was synthesized and copolymerized with PBAT’s main chain, creating an intrinsically UV-stable PBAT. The UV-stable PBAT was evaluated for UV resistance, mechanical performance, and durability through accelerated aging and solvent extraction tests. The results demonstrated that UV-stable PBAT exhibited exceptional light stabilization effects, with no detectable UV absorber leaching in ethanol even after 114 h, whereas PBAT blends lost nearly 90% of UV-0 within 24 h. Furthermore, UV-stable PBAT maintained 67.1% tensile strength and 48.8% elongation at break after aging, which exhibited the best mechanical retention performance. Even when subjected to solvent extraction, the 42.6% tensile strength retention outperformed the PBAT blends. This innovative chemical modification overcomes the limitations of additive-based stabilization, offering improved durability, compatibility, and performance in outdoor applications. Our research provides key insights into the fundamental properties of PBAT films for UV resistance, demonstrating their potential for use in demanding fields such as agricultural films.

## 1. Introduction

With the increasing worldwide concern about the environmental problems caused by traditional polymeric materials that are difficult to degrade in the natural environment, biodegradable polymers have attracted significant attention from academic and industrial fields in recent decades [1,2,3]. Among these polymers, poly(butylene adipate-co-terephthalate) (PBAT), a promising biodegradable aliphatic–aromatic copolyester, exhibits balanced mechanical properties and biodegradability, making it a key material to solve white pollution [4,5].

PBAT is a hydrolytically and biologically degradable polyester that requires effective stabilization against ultraviolet (UV) radiation to ensure reliable performance during its functional lifetime in agricultural mulch [6]. As biodegradable mulch films are deployed under continuous outdoor exposure throughout an entire growing season—ranging from several weeks to months—they must retain sufficient mechanical integrity to fulfill their protective role. Without UV stabilization, PBAT is prone to premature photodegradation, which leads to early embrittlement, cracking, and mechanical failure, ultimately compromising its functionality as a mulch film [7]. Therefore, enhancing the UV resistance of PBAT is critical to ensuring its functional stability and performance throughout its service life under complex outdoor environmental conditions.

Traditional methods to enhance the UV resistance of polymers with the incorporation of UV stabilizers have been extensively studied [8,9,10,11,12]. These stabilizers include small-molecule compounds such as UV absorbers, free radical scavengers, and quenchers, as well as inorganic UV shields like ZnO [13], TiO_2_ [10], and carbon black [14]. Incorporating these stabilizers into the polymer matrix during the melting process is a common approach to extend the service life of polymers. Nevertheless, the addition of these stabilizers often faces challenges that they are prone to migration under environmental conditions like exposure to water, oil, or other solvents, ultimately reducing their effectiveness [15].

Extensive research has been conducted to address the migration issues of light stabilizers [16,17,18,19]. For example, chemically immobilizing benzophenone-based UV absorbers onto silica nanoparticles, followed by their dispersion within the PBAT matrix, has demonstrated significant improvements in migration resistance and thermal stability [20]. New methods, such as developing hybrid materials by immobilizing UV absorbers (UV-As) onto inorganic supports or intercalating them into layered double hydroxides [17,18], have been explored to enhance sustainability and performance. Additionally, novel UV absorbers with large molecular weights have been synthesized to prevent migration and improve thermal stability [21].

However, while such modified light stabilizers enhance UV resistance performance, they cannot be uniformly dispersed within the polymer matrix and are prone to migration loss in thin film products, which prevents them from providing long-term light stabilization effects. Advancements in polymer chemistry have enabled the functional tailoring of PBAT-based polyesters through the copolymerization of diverse fourth monomers into the PBAT backbone, allowing for the precise modulation of material properties to meet specific application requirements. For instance, disulfide-containing monomers have been introduced to PBAT to enable redox-responsive degradation [22], while oxalic acid substitution has led to polyesters with enhanced hydrolytic rates and water barrier performance [23]. The incorporation of flexible PEG chains has improved hydrophilicity and transparency by reducing crystallinity [24], and glycolic acid copolymerization has yielded materials with higher tensile strength and improved seawater degradability [25]. These strategies collectively highlight the versatility of PBAT functionalization through molecular design.

In this work, a polymerizable UV absorber 2-hydro-4-(2,3-epoxypropoxy) benzophenone (HEPBP) containing an epoxy group which replaces the hydroxyl group on the fourth position of the benzene ring was successfully synthesized. Subsequently, varying amounts of HEPBP were copolymerized with 1,4-butanediol, terephthalic acid, and adipic acid, leading to the formation of an intrinsically UV-stable PBAT with the UV absorber covalently bound to the PBAT chains. The incorporation of a UV absorber into PBAT was achieved, and the photostability of neat PBAT and UV-stable PBAT containing the bound UV absorber was compared under accelerated aging tests. A solvent extraction test was also conducted to evaluate the migration behavior of the UV absorber in UV-stable PBAT and PBAT blended with UV-0. This approach not only addresses the migration issues associated with conventional light stabilizers during PBAT processing and blending but also offers a novel strategy for enhancing PBAT’s photostability through chemical synthesis.

## 2. Materials and Methods

### 2.1. Materials

2,4-dihydroxy benzophenone (UV-0, 99%), epichlorohydrin (99%), sodium hydroxide (99%), terephthalic acid (TPA), and adipic acid (AA) were provided by Shanghai McLean Biochemical Technology Co., Ltd. (Shanghai, China). 1,4-butanediol (1,4-BDO) was supplied by Shanghai Titan Technology Co., Ltd. (Shanghai, China). Tetrabutyl titanate (TBT, 99.5%) as a catalyst was obtained from Zancheng (Tianjin Technology Co., Ltd., Tianjin, China). All chemicals and solvents were used as received without further purification.

### 2.2. Synthesis of Reactive Light Stabilizer

The reactive light stabilizer was synthesized according to our group’s previous work reported in previous studies [26]: UV-0 (0.5 mol) was added to an aqueous sodium hydroxide solution (0.5 mol) in deionized water, with stirring and heating applied until a homogeneous solution was obtained. Epichlorohydrin (1.5 mol) was then introduced dropwise, and the reaction mixture was maintained at 60 °C under continuous stirring for 4 h until phase separation was observed. The crude product was washed with deionized water to pH ≈ 7 and subsequently concentrated by rotary evaporation to remove residual solvents. The resulting product was precipitated by the addition of methanol, yielding pale yellow crystals. These were collected by vacuum filtration and dried to afford 2-hydro-4-(2,3-epoxypropoxy) benzophenone (HEPBP) as the final product.

### 2.3. Synthesis of UV-Stable PBAT

**Scheme 1:**
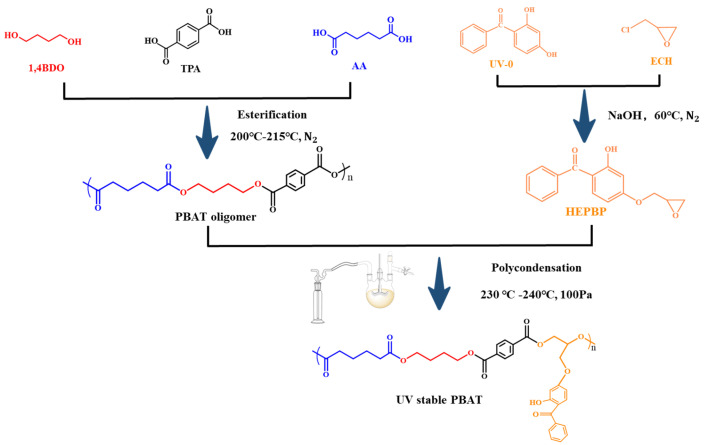
Synthesis route for UV-stable PBAT copolymers.

A mixture of terephthalic acid (TPA, 18.7 g, 112.5 mmol), 1,4-butanediol (27 g, 300 mmol), and varying amounts of HEPBP (0–0.34 g) was introduced into a 250 mL three-neck flask equipped with a condenser and mechanical stirrer. The esterification reaction was carried out at 215 °C under a nitrogen atmosphere for 3 h. Afterwards, acrylic acid (AA, 20 g, 134.5 mmol) and titanium butoxide (TBT, 30 μL) were added, and the temperature was gradually decreased to 180 °C. Stirring was continued under nitrogen for an additional 3 h until 90% of the theoretical water yield had been collected, indicating the completion of esterification. The polycondensation was then initiated by connecting the system to a vacuum pump via a cold trap, reducing the pressure to below 100 Pa, and heating to 230 °C for 3 h. The synthesized UV-stable PBAT was first dissolved in chloroform and then precipitated with cold methanol to obtain the final purified product. The synthesized PBAT was labeled PBAT, PBAT-0.2%, PBAT-0.3%, PBAT-0.4%, and PBAT-0.5%, corresponding to the addition of 0%, 0.2%, 0.3%, 0.4%, and 0.5% of HEPBP, respectively. The process was shown in Scheme 1.

### 2.4. Preparation of PBAT + UV-0 Blends

To avoid potential interference from any unknown additives present in commercial polymers, neat PBAT was first prepared in our laboratory. Then, PBAT (50 g) and UV-0 (0.15 g) were processed in an internal mixer at 160 °C and a rotor speed of 30 rpm. The mixture was blended for 5 min until the torque stabilized, at which point the PBAT blend was removed and allowed to cool. The prepared blends were denoted as PBAT+0.3% and PBAT+0.5% according to the addition amount of 2,4-dihydroxy benzophenone 0.3% and 0.5%, respectively. This preparation ensured that the UV resistance of the PBAT blends could be only attributed to the addition of UV-0.

### 2.5. Characterization

The number-average (M_n_) and weight-average (M_w_) molecular weights of PBAT polyesters were determined using gel permeation chromatography (GPC) at 35 °C. The analysis was performed with a Waters 2414 system equipped with three detectors: Waters 2414, Wyatt Treos II, and Wyatt ViscoStar III. Chloroform served as the mobile phase, with an elution flow rate of 1 mL/min.

The intrinsic viscosities $\left[\eta \right]$ were tested at 25 °C by an ubbelohde viscometer. The samples were dissolved in solvents of tetrachloroethane/phenol (50 wt%/50 wt%) to achieve a homogeneous solution at a concentration of 0.5 g/dL. Intrinsic viscosity was obtained as Equations (1) and (2):

(1) $$\eta_{sp}=\frac{t-t_{0}}{t_{0}}$$

(2) $$\left[\eta \right]=\frac{\sqrt{1+1.4\eta_{sp}}-1}{0.7c}$$

where $\eta_{sp}$ is the specific viscosity, *t*_0_ is the outflow time of pure solvent, *t* is the outflow time of solution, and *c* is the concentration of the solution [27].

UV–vis spectra were recorded on a Hitachi U-3010 spectrophotometer (Hitachi, Tokyo, Japan) equipped with an integrating sphere for diffuse reflection in the wavelength range of 200–600 nm.

The chemical compositions of HEPBP and PBAT samples were confirmed by ^13^C-NMR and ^1^H-NMR spectroscopy, recorded on a JNM-ECA600 NMR (JEOL, Tokyo, Japan) spectrometer with deuterated chloroform as the solvent. FT-IR spectroscopy was performed on a Thermo Scientific-Nicolet 6700 (Thermo Fisher Scientific, Waltham, MA, USA) in reflection mode, with the wavenumber ranging from 4000 cm^−1^ to 500 cm^−1^.

Tensile tests were conducted at 25 °C using a Jinjian UTM-1432 universal testing machine with a crosshead speed of 50 mm/min. Test specimens, shaped like dumbbells and measuring 25 mm in length, 4 mm in width, and 2 mm in thickness, were prepared by hot pressing at 160 °C under 30 MPa for 2 min. Each sample was tested at least five times, and the results were reported as average values.

The thermal behavior of the samples was analyzed using differential scanning calorimetry (TA-DSC250) under a nitrogen atmosphere. The procedure involved an initial heating from 30 °C to 160 °C at a rate of 10 °C/min, followed by a 3 min isothermal hold at 160 °C to erase prior thermal history. The samples were then cooled to −50 °C at the same rate and held for 3 min before undergoing a second heating cycle up to 160 °C at 10 °C/min.

Wide-angle X-ray diffraction (WAXD) measurements were conducted at room temperature using a Bruker D8 Advance diffractometer with Cu-Kα radiation. Scanning was carried out over a 2θ range of 5° to 35°, with a step size of 0.02° and a scanning rate of 4°/min.

Rheological properties were assessed using an Anton Paar Physica MCR301 rheometer equipped with an 8 mm diameter parallel-plate geometry and a 1 mm gap. The tests were performed at 160 °C using dynamic frequency sweep mode at a constant strain of 1%, with angular frequencies ranging from 100 to 0.1 rad/s.

Enzymatic degradation experiments were conducted in a shaking water bath maintained at 37 °C. The experimental setup involved immersing the samples in 8 mL of phosphate-buffered solution containing cutinase, while the control group used the same volume of buffer without the enzyme. At predetermined time intervals, the films were removed, thoroughly washed, dried to a constant weight, and weighed to assess degradation. The degradation level was estimated from the percentage of weight loss according to Equation (3):

(3) $$\begin{array}{c} \mathrm{weight}\;\mathrm{loss}\left(\%\right)=\frac{m_{0}-m_{t}}{m_{0}}\times 100\% \end{array}$$

where $m_{0}$ is the original weight, and $m_{t}$ is the weight after degradation.

### 2.6. Accelerated Aging Test

All PBAT, UV-stable PBAT, and PBAT+UV-0 blends were molded into dumbbell-shaped specimens with dimensions of 25 mm in length, 4 mm in width, and 2 mm in thickness through hot pressing at 160 °C and 30 MPa for 2 min. An accelerated weathering tester (QUV, Q-Lab, Westlake, OH, USA) was employed to assess the UV durability of the functional materials under these conditions (65 °C, 0.70 W/m^2^, 340 nm).

### 2.7. Solvent Extraction Test

The migration behavior of the UV absorber from all UV-stable PBAT and PBAT+UV-0 blends was evaluated at room temperature using ethanol as the extraction solvent. At specific time intervals, samples of the extraction solution were collected and analyzed. A UV–visible spectrophotometer was employed to characterize the solvent and quantify the amount of UV absorber dissolved in it, thereby assessing the migration resistance of the UV absorber within the PBAT matrix.

### 2.8. In Situ FTIR Accelerated Aging Evaluation

CO_2_ production during the photo-oxidation of PBAT was determined using the in situ FTIR system developed in our laboratory. A circular PBAT sample was sealed in a 30 mL quartz cell affixed to an FTIR spectrometer (Nicolet IS50, Thermo Fisher Scientific Inc., Waltham, MA, USA). The cell was purged with pure air (without CO_2_) for 20 min at varying humidity levels to ensure a constant background. Subsequently, the test began, capturing the transmission FTIR spectra (32 scans, 4000−1111 cm^−1^, 4 cm^−1^ resolution) of the gaseous phase inside the cell every 5 min. For the first 30 min, the sample was maintained at 30 °C without UV exposure. Subsequently, the temperature was raised to 60 °C, and UV irradiation was applied at an intensity of 60 mW/cm^2^ (@365 nm). A xenon lamp (model 66984300XF-R1, Newport, Irvine, CA, USA) fitted with an AM 1.5 solar filter (81094, Newport, Irvine, CA, USA) was used to simulate terrestrial sunlight across the 300–800 nm wavelength range. After 210 min of exposure, both the heating and UV light were turned off. The relative concentration of CO_2_ was quantified by integrating the area under its absorption peaks between 2433 cm⁻^1^ and 2253 cm⁻^1^, using baseline correction without accounting for PBAT.

## 3. Analysis and Discussion

### 3.1. Synthesis and Characterization of HEPBP UV Absorber

By using thin-layer chromatography (TLC) to track the reaction process as seen in Figure S2, the best modification process has been determined for maximum yield and minimum by-products. The mixture of products was separated by column chromatography on silica for further chemical structure analysis.

**Figure 1:**
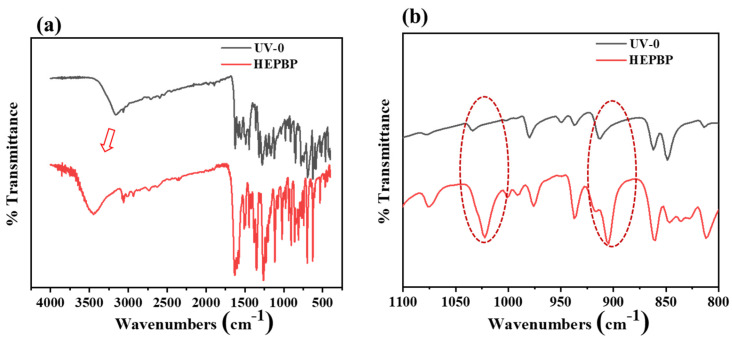
FTIR spectra of (**a**) UV-0 and HEPBP, (**b**) fingerprint peak around 1100–800 cm^−1^.

Based on Figure 1, the comparison of the FTIR spectra of UV-0 before and after reaction with ECH reveals notable changes. After chemical modification, the O–H stretching vibration at 3423.5 cm⁻^1^ is significantly reduced compared to that in UV-0, as seen the arrow in Figure 1a. Additionally, new absorption peaks appear at 3061 cm⁻^1^ and 2738 cm⁻^1^, corresponding to the C–H stretching vibrations of the methylene groups in the epoxy moiety. The characteristic absorption peak of the carbonyl bond in benzophenone is observed at 1627 cm⁻^1^, while the epoxy C–O–C stretching vibration peaks emerge at 1022 cm⁻^1^ and 905 cm⁻^1^, confirming the formation of ether bonds and the introduction of epoxy groups on UV-0 (an epoxy-hydroxypropyl group replaces the hydroxyl group on the fourth position of the benzene ring), as seen the circles in Figure 1a.

**Figure 2:**
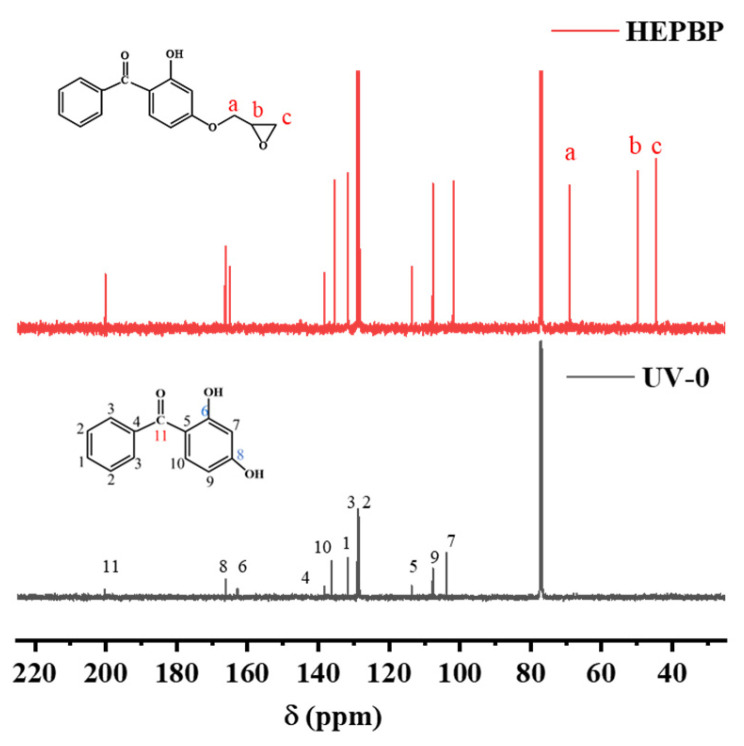
^13^C- NMR spectrum of UV-0 and HEPBP.

The 13 carbon atoms of UV-0 are in 11 different chemical environments as indicated in the ^13^C-NMR spectrum in Figure 2. Specifically, there are six methine carbon signals (103.6, 107.5, 128.3, 128.8, 131.5, 136.1 ppm) and five quaternary carbon signals (113.5, 138.1, 166.1, 162.7, 200.2 ppm). Notably, due to the conjugation effect, the signal of the carbonyl carbon shifts downfield, appearing at 200.2 ppm. The signals at 166.1 ppm and 162.7 ppm are assigned to the carbons connected to the ortho-OH and para-OH groups, respectively. The signals at 128.8 and 128.3 ppm are characteristic of a symmetrically monosubstituted aryl group. After modification, three additional C atom characteristic peaks were observed as labeled: C(a), C(b), and C(c). It is noted that the C8 signal shifted from 162.7 ppm to 164.9 ppm due to the replacement of the para-OH group with the epoxy-hydroxypropyl group.

Moreover, from the ^1^H-NMR spectra of HEPBP (Figure S1), the integral analysis shows absorption peaks in the 6.47–7.61 ppm range corresponding to hydrogen atoms (H2-H6) connected to the benzene rings, while the peak at 12.63–12.66 ppm represents the hydrogen atoms on the phenolic hydroxyl groups (H1). The peak at 12.75–4.32 ppm assigns hydrogen atoms to the epoxy group (H7-H9). The ratio of these peaks reflects the relative amounts of hydrogen atoms in different chemical environments. Both the ^13^C-NMR and ^1^H-NMR spectra demonstrated HEPBP with specific reactive functional groups.

**Figure 3:**
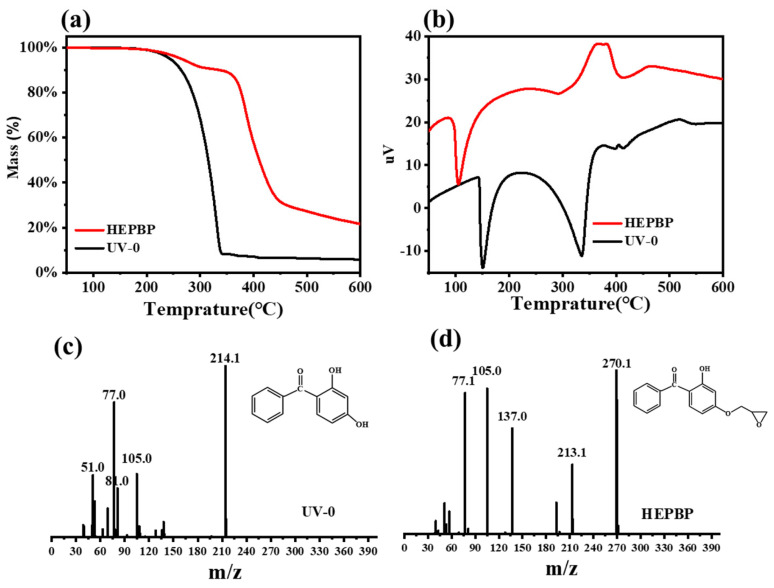
(**a**) TGA and (**b**) DTA of UV-0 and HEPBP, (**c**,**d**) GC-MS of UV-0 and HEPBP.

Thermogravimetric analysis (TGA) and differential thermal analysis (DTA) values were obtained for UV-0 and HEPBP at a heating rate of 20 °C/min under nitrogen. Figure 3b shows the thermal decomposition temperature. UV-0 exhibited endothermic transition but no significant mass loss at 148 °C and was similar to HEPBP at 98 °C. So those were the melting points. The other endothermic transition of UV-0 was at 320 °C, and the residual mass was nearly 0. That was the boiling point of UV-0. However, an exothermic transition of HEPBP is shown at around 380 °C, where thermal decomposition reaction occurred. In addition, the GC-MS displayed an m/z value of 271.1Da, suggesting the molecular formula C_16_H_14_O_4_ was obtained, while UV-0 C_13_H_10_O_3_ displayed a value of 214.1, as shown in Figure 3c,d.

**Figure 4:**
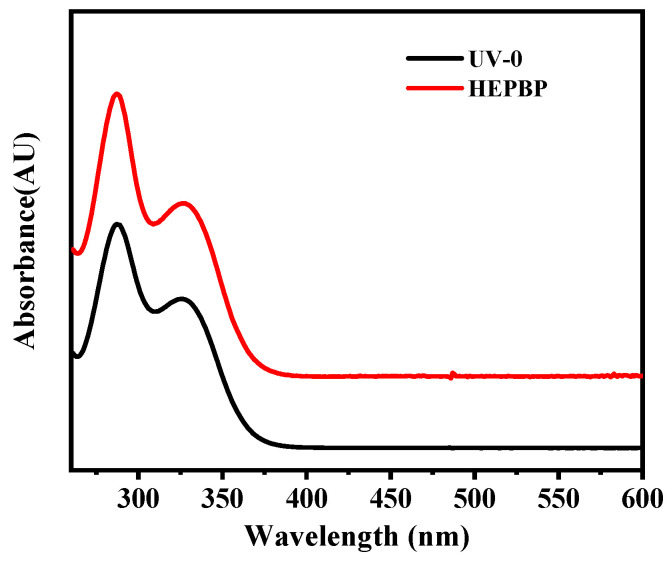
The UV absorption spectra of UV-0 and HEPBP.

As shown in Figure 4, UV-0 and HEPBP exhibit similar absorption characteristics, with both displaying two strong absorption bands in the UV region (200–400 nm). The maximum absorption wavelength λ_max_ for both compounds appears in the first band, between 280 and 290 nm. Compared to UV-0, the absorptivity coefficient ε of HEPBP is significantly higher, indicating that HEPBP has a stronger UV absorption capability than that of UV-0 (see Table 1 and the calibration curves in Figure S3). Meanwhile, a slight red shift in the λ_max_ of the HEPBP is also observed. This may be attributed to the electron-donating properties of the linker. When an epoxy group is introduced into the side chain of benzophenone, the electron cloud density of the entire conjugated system increases, leading to a decrease in the π-π* transition energy. Consequently, the absorption wavelength shifts to the red, and the absorptivity coefficient increases.

Based on the above analysis, the HEPBP absorber with the anticipated chemical structure was successfully obtained through thin-layer chromatography separation, enhancing its UV absorption efficiency. Subsequently, the HEPBP UV absorber was incorporated during the polymerization of PBAT to further investigate its incorporation into the PBAT main chain and its impact on the performance of PBAT copolymers.

### 3.2. Molecular Chemical Structure of UV-Stable PBAT

**Figure 5:**
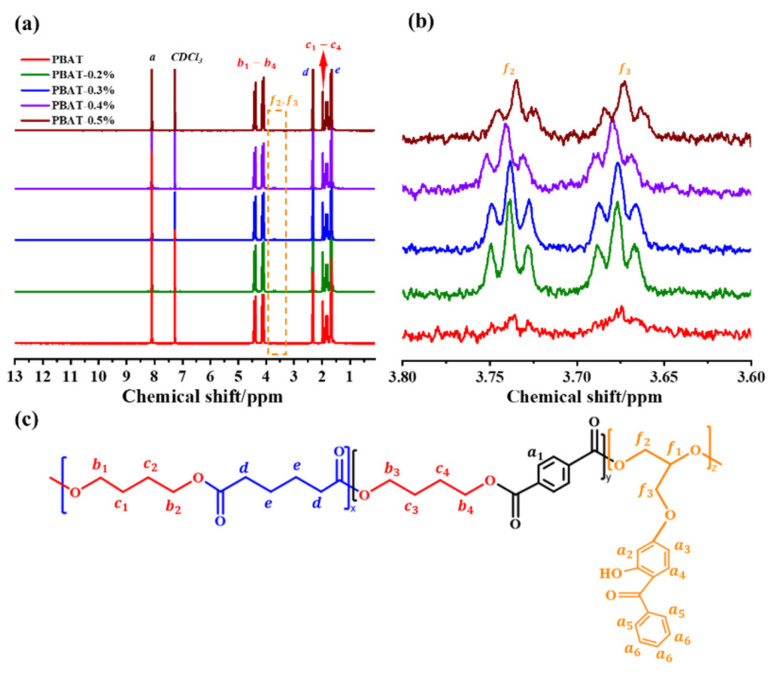
(**a**) ^1^H-NMR spectra of functional and neat PBAT copolymers; (**b**) enlargement of chemical shifts at 3.60–3.80 ppm; (**c**) chain structures of the UV-stable PBAT copolymers.

The molecular weight, intrinsic viscosity, copolymer composition, and sequence length of the copolyesters are summarized in Table 2. The ^1^H-NMR spectra of the copolyesters are shown in Figure 5. All the PBAT copolymers possess Mw values of 68.3–70.2 kDa. The intrinsic viscosities of UV-stable PBAT are in the range of 1.32−1.40 dL/g, which are similar to those of neat PBAT.

To ensure that the copolyesters have good mechanical properties and biodegradability, the feed ratio of TPA and AA is controlled at 45:55. The molar percentages of TPA ($n_{TPA}$), AA ($n_{AA}$), and HEPBP ($n_{UV}$) were calculated using Equations (4)–(6).

(4) $$n_{TPA}=\frac{I_{a}}{I_{a}+I_{d}+I_{f2}+I_{f3}}\times 100\%$$

(5) $$n_{AA}=\frac{I_{d}}{I_{a}+I_{d}+I_{f2}+I_{f3}}\times 100\%$$

(6) $$n_{UV}=\frac{I_{f2}+I_{f3}}{I_{a}+I_{d}+I_{f2}+I_{f3}}\times 100\%$$

In addition, the number-average sequence length of BA ($L_{BA}$), BT ($L_{BT}$), and randomness (R) are calculated using Equations (7)–(9). The value of R can reflect the randomness of connection. In this work, an R close to 1 indicated that all the copolymers had a random sequence structure.

(7) $$L_{BA}=\frac{I_{b_{2}}}{I_{b_{3}}}+1$$

(8) $$L_{BT}=\frac{I_{b_{1}}}{I_{b_{3}}}+1$$

(9) $$R=\frac{1}{L_{BA}}+\frac{1}{L_{BA}}$$

The ^1^H-NMR spectrum of UV-stable PBAT with different amounts of HEPBP is shown in Figure 5a. Due to the similar chemical environments, the characteristic peaks of hydrogen atoms on HEPBP largely overlap with the characteristic peaks of hydrogen atoms in the PBAT main chain. However, a weak characteristic peak signal was detected at 3.60–3.80 ppm, corresponding to the characteristic absorption peak of Hf, indicating the incorporation of the epoxy group into the molecular main chain. Additionally, the characteristic absorption peaks of the epoxy functional group were observed in the infrared spectrum in the fingerprint region of 850–1300 cm⁻^1^. By comparing the ^1^H-NMR and FTIR spectra of neat PBAT and UV-stable PBAT (Figure S4), it is confirmed that HEPBP is covalently bonded within the UV-stable PBAT chain.

### 3.3. Thermal and Crystallization Properties

The crystal structure of the UV-stable PBAT was characterized using XRD, as shown in Figure S5. All the PBAT copolymers exhibited five main peaks at around 2θ = 16.1°, 17.3°, 20.1°, 22.8°, and 24.5°, corresponding to the (011), (010), (111), (100), and (101) reflection planes of PBT-like crystals. No peaks corresponding to the crystal structure of PBA [28] were observed in this study, indicating that only PBT crystals were present in PBAT. Furthermore, compared to pure PBAT, all UV-stable PBAT samples exhibit similar diffraction peaks at 28.3° and 31.5° for the (104) plane of the β-form of PBT [29]. This β form was an unstable crystalline structure that undergoes a phase transformation into the stable α crystal upon secondary heating. This phase transformation can be detected in DSC testing, where a minor endothermic peak is observed around 50–60 °C.

**Figure 6:**
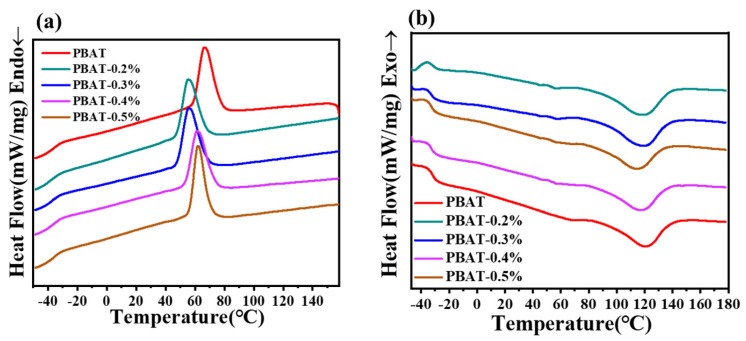
DSC curves of (**a**) cooling, and (**b**) second heating of neat and UV-stable PBAT samples.

The melting behavior of neat PBAT and UV-stable PBAT was characterized by DSC, and the thermal transition data are summarized in Table 3. According to the discussion of the crystalline structure, only BT segments in PBAT can crystallize at a certain sequence length, as the BA segments are randomly linked between the BT segments. The DSC results (Figure 6) showed that the crystallization temperature shifts to a lower value, indicating that the HEPBP side chains hinder the orderly arrangement of molecular chains and cannot be incorporated into the lattice, acting as defect points that reduce crystallization ability, thus requiring a higher degree of supercooling for crystallization. Lower crystallization enthalpy and melting enthalpy are observed in UV-stable PBAT, indicating a reduced degree of crystallinity.

### 3.4. Mechanical Property

The UV-stable PBAT copolyester incorporated with UV stabilizer side chains exhibits a notable balance of strength and flexibility, derived from its unique chain structure. The crystalline BT hard segments impart strength to the copolymer, while the flexible BA segments confer ductility. The stress–strain curves for these PBAT copolymers exhibit high elongation at break and partially reversible deformation. Additionally, prominent strain hardening is observed, likely due to strain-induced crystallization and chain alignment under tensile load.

**Figure 7:**
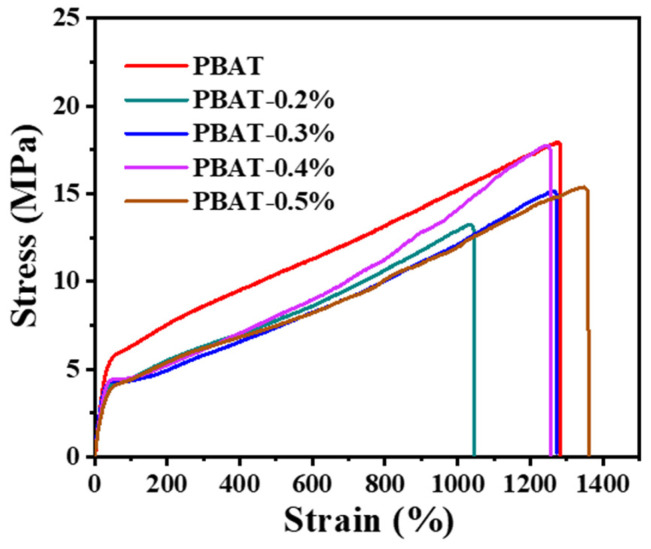
Typical stress–strain curves of neat and UV-stable PBAT.

As shown in Figure 7 and based on the details in the mechanical property data (Table 4), grafting UV stabilizer side chains onto PBAT leads to a reduction in mechanical performance relative to neat PBAT, as evidenced by decreases in both tensile strength and elongation at break. This decline is primarily attributed to the rigid benzene ring structure of the UV stabilizer side chains, which disrupts the ordered crystallinity and stability of the BT chain segments, thereby lowering the overall crystallinity of the copolyester.

The reduced crystallinity provides a higher elongation potential, yet the presence of the rigid benzene rings weakens inter-chain entanglement and increases the distance between adjacent backbone chains. This increased spacing diminishes intermolecular interactions, further contributing to a reduced elongation at break.

### 3.5. Rheological Properties

In the low-frequency region, PBAT-0.4% and PBAT-0.5% exhibit higher complex viscosities compared to neat PBAT with similar molecular weights. This phenomenon can be attributed to the introduction of incorporated side chains, leading to enhanced molecular entanglements, primarily involving interactions between the main chains and branched side chains. Additionally, as the frequency increases, UV-stable PBAT demonstrates more significant shear-thinning behavior. This is similar to the results observed in systems with aliphatic branched side chains [30,31]. However, due to the rigid aromatic benzophenone structures in the grafted side chains, the entanglement capability is lower than that of aliphatic structures, resulting in a more pronounced shear-thinning response.

**Figure 8:**
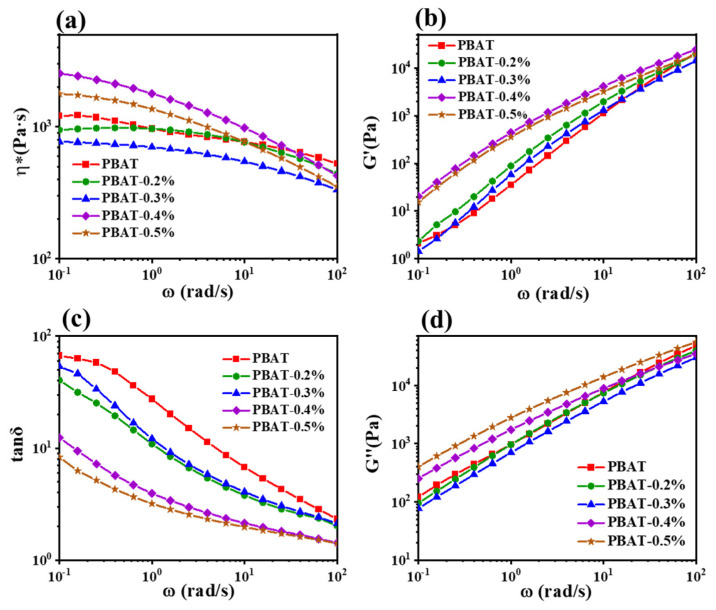
Rheology results: (**a**) complex viscosity η*; (**b**) storage modulus G′; (**c**) loss factor tan δ; (**d**) loss modulus G″ of neat and UV-stable PBAT.

The enhanced molecular entanglement within UV-stable PBAT contributes to higher storage modulus (G’) and loss modulus (G”) values compared to neat PBAT, particularly in the low-frequency region. Loss factor tanδ is defined as the ratio of $G^{\prime \prime }$ to $G^{\prime }$. tanδ = 1 represents the transition from the viscous to elastic responses in the polymer melt. A smaller tanδ indicates a smaller phase difference between the strain and stress produced by the melt, corresponding to a higher elastic response. As depicted in Figure 8, the introduction of benzophenone-based side chains into PBAT results in a corresponding decrease in the tanδ value at the same frequency, indicating an improvement in the elasticity of the PBAT melts.

### 3.6. Enzymatic Hydrolysis

**Figure 9:**
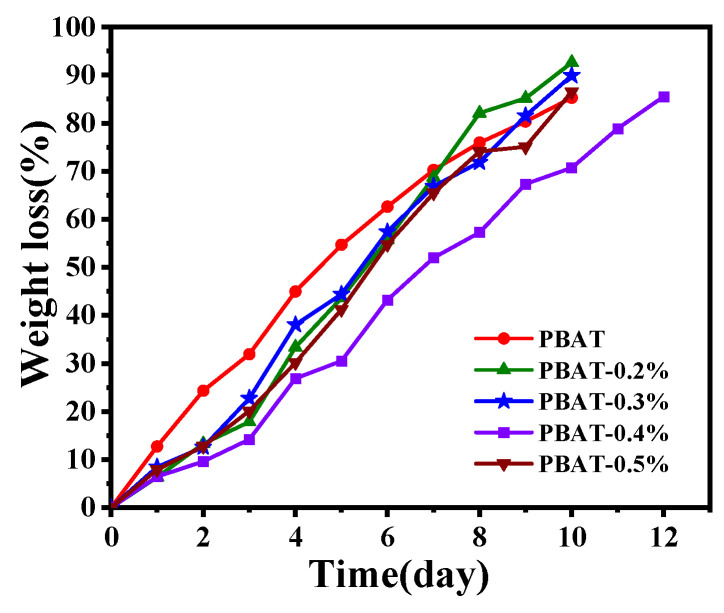
Degradation of neat and UV-stable PBAT in cutinase solution.

The initial stage of biodegradation is hydrolysis, where macromolecular polymers are degraded into smaller oligomers under the catalysis of enzymes. In this study, cutinase was selected for the enzymatic hydrolysis test to evaluate the impact of the HEPBP side chain on the degradation rate of PBAT. The hydrolysis experiments for functional and neat PBAT samples were conducted in phosphate-buffered solution with cutinase at 37 °C, using a cutinase concentration of 0.6 mg/mL. Degradation was induced by the cleavage of ester linkages, leading to the formation of oligomeric and monomeric products, which ultimately caused weight loss. Figure 9 illustrates the in vitro enzymatic hydrolysis of PBAT. As expected, the introduction of the HEPBP side chain slowed the enzymatic hydrolysis rate of PBAT, mainly due to the steric hindrance effect of the branched chains, which impeded the recognition and binding of cutinase’s active pocket to the characteristic groups of PBAT.

### 3.7. In Situ FTIR Accelerated Aging Evaluation

**Figure 10:**
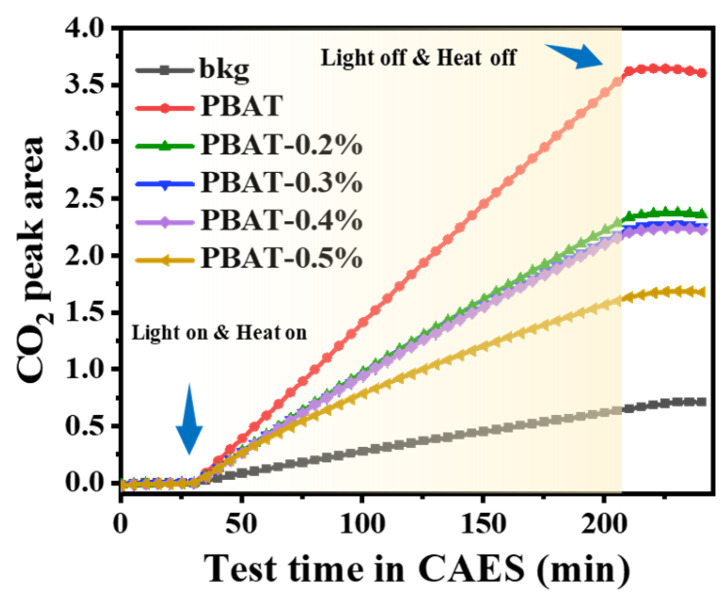
In situ FTIR aging evaluation of neat and UV-stable PBAT.

In situ FTIR aging assessment was conducted to compare the UV resistance of UV-stable PBAT with that of neat PBAT. Samples were exposed to simulated solar radiation at an irradiance of 60 mW/cm^2^ and a temperature of 60 °C for a duration of 3 h, and their aging resistance was evaluated by measuring CO_2_ release. The effectiveness of this advanced characterization method has been validated in previous studies [32,33,34]. After background correction, the CO_2_ emission data is presented as shown in Figure 10.

The data reveals that all samples exhibit CO_2_ release at a consistent release rate, showing no abrupt fluctuations. Notably, the UV-stable PBAT demonstrates significantly enhanced UV resistance, and the degree of UV resistance improves as the content of the UV-0 modification increases. Among the samples, PBAT with 0.2–0.4% UV stabilizer shows similar levels of CO_2_ release, suggesting comparable UV resistance in the groups. However, the PBAT-0.5% sample exhibits the lowest CO_2_ release over the same testing period, indicating superior UV resistance relative to the PBAT-0.2%, PBAT-0.3%, and PBAT-0.4% samples. This phenomenon aligns with the molecular chain structure, which incorporates an increased content of modification UV-0.

These results underscore the effectiveness of the UV-0 modification in UV-stable PBAT, providing enhanced UV resistance with increasing stabilizer content contributing progressively to the PBAT copolymer’s durability under simulated environmental exposure.

### 3.8. Solvent Extraction Test

**Figure 11:**
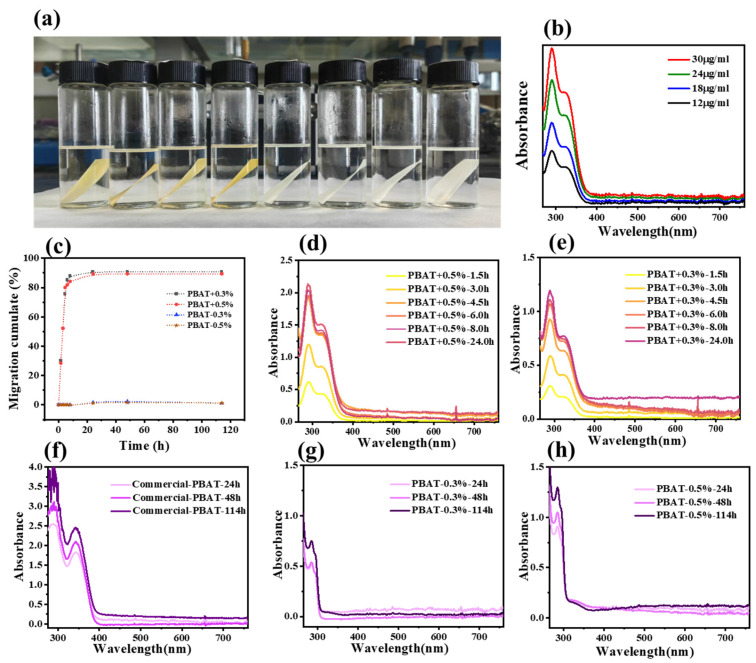
(**a**) Solvent extraction test of PBAT blends and UV-stable PBAT samples. (PBAT-0.2%, PBAT-0.3%, PBAT-0.4%, PBAT-0.5%, neat PBAT, PBAT+0.3%, PBAT+0.5%, and KF-PBAT, from left to right). (**b**) The UV spectra of UV-0 with different concentrations. (**c**) Percentage cumulation of the UV absorber migrating from sample films in ethanol. The UV spectra of the extraction solution with different extraction times: (**d**) PBAT+0.5%UV-0 blends, (**e**) PBAT+0.3%UV-0 blends, (**f**) commercial PBAT, (**g**) PBAT-0.3% copolymer, and (**h**) PBAT-0.5% copolymer.

UV-stable PBAT exhibits excellent UV resistance properties. The extractability and migration behavior of UV absorbers are crucial for long-term performance. In the solvent extraction test, the migration of UV absorbers from UV-stable PBAT- and PBAT-blend films containing UV-0 was monitored using UV-Vis spectroscopy. Considering the solubility of UV-0 and PBAT samples, ethanol was selected as the extraction solvent. The UV–visible absorption spectra of UV-0 at different concentrations (Figure 11a) were used to establish a calibration curve by plotting the absorbance against concentration according to the Beer–Lambert law. Based on this calibration curve (Figure S2), a comparative solvent extraction analysis was conducted between commercial PBAT (KF-PBAT), PBAT blends with 0.3% and 0.5% UV-0 loading (PBAT+0.3%, PBAT+0.5%), and UV-stable PBAT copolymer with the same content (PBAT-0.3%, PBAT-0.3%). A sharp increase in absorbance at 327 nm indicates that UV-0 is readily extracted from the PBAT blends over time, reaching nearly 90% extraction within 24 h. This rapid extraction is attributed to the weak physical interactions between UV-0 and the PBAT matrix, which facilitate solvent-driven migration of UV-0 out of the PBAT blends. During the initial hours, a considerable amount of UV-0 is located near the surface of the sample film, where a pronounced concentration gradient between the film and the extraction solvent drives UV-0 migration, resulting in a sharp increase in extraction rate.

In contrast, the UV-stable PBAT copolymer exhibits markedly lower extraction rates, with no discernible UV absorption peak at 330 nm observed even after 114 h, indicating that the UV stabilizer remains linked in the main chain and will not migrate out of the PBAT matrix in the solvent environment. These results demonstrate that the covalent bonds between the PBAT chains and the UV-stabilizing groups will not be extracted. Furthermore, when compared to commercial PBAT blended with nanohybrid UV absorbers, a strong UV absorption peak at 321 nm is observed under identical extraction conditions, suggesting that the UV stabilizer is rapidly leached from the PBAT matrix in solution. The extraction rate of UV-stable PBAT was also lower than those reported in the literature [18]. This analysis highlights the superior solvent resistance of UV-stable PBAT, suggesting its potential for enhanced durability and UV resistance under outdoor environmental conditions.

### 3.9. Accelerated Aging Test

The UV resistance performance of UV-stable PBAT and PBAT blends was evaluated through tensile tests after 20 days of accelerated UV aging. PBAT-1 and PBAT-2 represent UV-stable PBAT and PBAT blends before solvent extraction, respectively. PBAT-3 and PBAT-4 represent UV-stable PBAT and PBAT blends after solvent extraction. PBAT-5 corresponds to neat PBAT.

**Figure 12:**
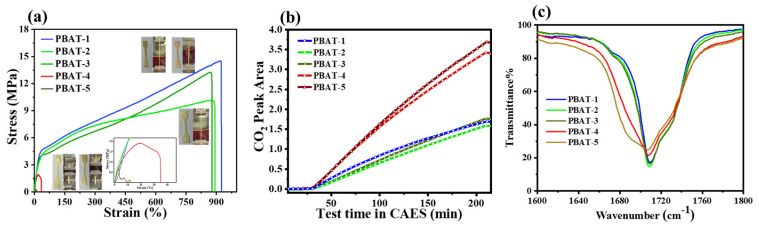
(**a**) Stress–strain curves of PBAT blends and UV-stable PBAT after accelerated aging test and (**b**) in situ FTIR aging evaluation of PBAT blends and UV-stable PBAT before and after solvent extraction test. (**c**) FTIR in 1600–1800 cm^−1^ of PBAT blends and UV-stable PBAT before and after solvent extraction test.

The solvent-extracted samples, PBAT-3 and PBAT-4, were subjected to in situ FTIR aging assessment, with the corresponding curves shown in Figure 12. UV-stable PBAT (PBAT-3) retained a significant level of UV resistance, indicating that its UV resistance remains effective even after solvent extraction. In contrast, the PBAT blend (PBAT-4) exhibited photo-aging behavior comparable to neat PBAT, highlighting that the leaching of light stabilizers from the matrix led to a substantial loss of UV protection in the PBAT system.

Following accelerated aging, PBAT-5 specimens exhibited severe surface cracking and brittle fracture during tensile testing, resulting in the complete loss of mechanical strength and rendering further mechanical evaluation infeasible. In contrast, PBAT-1 and PBAT-2, which incorporate light-stabilizing components, retained superior mechanical properties after aging. For PBAT-1, in which HEPBP covalently bonded to the PBAT main chain, the tensile strength decreased from 21.6 MPa to 14.5 MPa, while the elongation at break reduced from 1884% to 920%. However, PBAT-2, which is a blend of PBAT and UV-0, exhibited comparatively lower mechanical strength retention under identical aging conditions (as seen in Table 5).

After 48 h of ethanol extraction, PBAT-3 and PBAT-4 showed notable changes in tensile behavior following accelerated aging, although no visible surface cracking was observed. The tensile strength of PBAT-3 decreased from 22.4 MPa to 10.1 MPa, while the elongation at break dropped from 1884% to 858%. Interestingly, PBAT-3 exhibited tensile behavior analogous to that of PBAT-1. In contrast, because of ethanol extraction, only a small amount of photostabilizer remains in the matrix of PBAT-4. Consequently, after 20 days of aging, PBAT-4 samples showed a dramatic reduction in mechanical strength despite the absence of surface cracking, resembling the photo-aging behavior of neat PBAT. The test highlights the crucial role of light stabilizers in mitigating the mechanical degradation of PBAT-based materials under UV exposure, as well as the impact of their extraction on long-term stability.

The Carbonyl Index (CI) is commonly used as a quantitative indicator to assess the aging degree of PBAT [35]. Specifically, the absorption band at 725 cm⁻^1^, corresponding to methylene C–H vibrations, is selected as the reference peak. The CI is calculated using the following formula to compare the photodegradation resistance before and after aging:

(10) $$\begin{array}{c} CI=\frac{A_{c=o\;}}{A_{C-H}}\times 100\%\; \end{array}$$

The CI change ratio after aging for PBAT-1 and PBAT-2 are 1.15 and 1.17, respectively, indicating similar UV resistance. After solvent extraction, the CI change ratio remains at 1.18 for PBAT-3 but increases to 1.45 for PBAT-4. As for PBAT-5, the neat PBAT sample exhibits the highest CI change after aging, reaching 1.55. As shown in Figure 12c, it can be observed that PBAT-4 and PBAT-5 exhibit a distinct shoulder at 1680 cm⁻^1^, which corresponds to the absorption of conjugated carbonyl groups, such as unsaturated ketones. This observation suggests that UV-stable PBAT demonstrates better UV resistance than the PBAT blends, while PBAT-4 and PBAT-5, lacking UV stabilizers, undergo accelerated aging and generate a wider variety of carbonyl species.

## 4. Conclusions

In summary, this study successfully developed an intrinsically light-stable PBAT by designing the molecular chain structure to incorporate the reactive UV absorber HEPBP through covalent bonding into the PBAT molecular chains via melt polycondensation, thereby addressing the issue of stabilizer migration in outdoor film applications. In situ FTIR accelerated aging evaluation and solution extraction experiments demonstrated that the intrinsically light-stable PBAT exhibited excellent light stability. The incorporation of side groups reduced the crystallization temperature and crystallinity of PBAT without altering its crystalline structure. The entanglement effect of the side groups enhanced the melt elasticity response. Furthermore, the UV-stable PBAT retained its biodegradability, although the introduction of the HEPBP side chain slowed the enzymatic hydrolysis rate, as the presence of the side groups affected the interaction between the active site and PBAT. The intrinsically UV-stable PBAT copolyesters, with their excellent UV resistance, hold significant potential for meeting the demands of complex outdoor environments, particularly in agricultural film applications.

## Figures and Tables

**Table 1:** Absorption parameters of UV-0 and HEPBP.

Sample	$\lambda_{max1}\;(nm)$	$\varepsilon_{1}$ $({ml\cdot \mu g\cdot cm}^{-1}$)	$\lambda_{max2}\;(nm)$	$\varepsilon_{2}\;({ml\cdot \mu g\cdot cm}^{-1})$
UV-0	287	0.0626	326	0.0419
HEPBP	290	0.0922	330	0.0566

**Table 2:** Molecular structures of the neat and UV-stable PBAT copolymers.

Sample	$n_{AA}$	$n_{PTA}$	$n_{UV}$	$L_{BA}$	$L_{BT}$	R	Mw (kDa)	Mn (kDa)	DPI	$\left[\eta \right]$ (dl/g)
PBAT	54.61	45.39	n.d	2.03	1.86	1.04	70.2	52.1	1.35	1.39
PBAT-0.2%	55.31	44.52	0.17	1.99	1.95	0.97	69.8	51.9	1.37	1.36
PBAT-0.3%	54.84	44.91	0.25	2.04	1.90	1.01	68.3	48.9	1.40	1.32
PBAT-0.4%	54.56	45.17	0.27	2.06	1.89	1.01	70.5	50.5	1.39	1.40
PBAT-0.5%	54.25	45.34	0.41	2.09	1.85	1.02	68.4	50.8	1.34	1.38

**Table 3:** Thermal parameters of neat and UV-stable PBAT.

Sample	$T_{c}\;({}^{\circ}C)$	$∆H_{c}\;(J/g)$	$T_{g}\;({}^{\circ}C)$	$T_{m}\;({}^{\circ}C)$	$∆H_{m}\;(J/g)$	$X_{c}\%$
PBAT	66.2	19.0	−32.0	120.4	16.2	14.2
PBAT-0.2%	55.2	17.9	−32.1	118.6	15.5	13.6
PBAT-0.3%	55.6	18.9	−33.2	118.8	15.4	13.5
PBAT-0.4%	61.3	18.5	−32.6	117.9	13.9	12.2
PBAT-0.5%	62.0	17.6	−33.0	114.3	13.0	11.4

**Table 4:** Mechanical property of neat and UV-stable PBAT.

Sample	E (MPa)	$\sigma_{0.2}$(MPa)	EF (%)	$\sigma_{b}$ (MPa)
PBAT	21.6 ± 0.9	5.9 ± 0.3	1296 ± 72	17.8 ± 2.1
PBAT-0.2%	18.9 ± 1.1	4.4 ± 0.2	1035 ± 91	13.2 ± 1.4
PBAT-0.3%	21.1 ± 0.6	4.9 ± 0.3	1272 ± 85	14.8 ± 1.7
PBAT-0.4%	18.1 ± 0.7	5.5 ± 0.4	1245 ± 81	17.8 ± 2.0
PBAT-0.5%	17.7 ± 0.6	5.1 ± 0.2	1347 ± 74	15.4 ± 1.9

**Table 5:** Mechanical property retention of neat and UV-stable PBAT after accelerated aging test.

Sample	$\sigma_{b}$(MPa)	EF(%)	$\sigma_{b}$	EF Retention
PBAT-1	14.5 ± 0.5	920 ± 48	67.1%	48.8%
PBAT-2	12.6 ± 0.6	874 ± 52	42.6%	47.7%
PBAT-3	10.1 ± 0.7	858 ± 65	45.1%	45.5%
PBAT-4	1.8 ± 0.3	35 ± 5	8.3%	2.1%
PBAT-5	0.1	3	<1%	<1%

## Data Availability

The raw data supporting the conclusions of this article will be made available by the authors on request.

## Supplementary Materials

The following supporting information can be downloaded at: https://www.mdpi.com/article/10.3390/polym17111567/s1, Figure S1. ^1^H- NMR spectrum of HEPBP. Figure S2. TLC profile of reaction of 2,4-dihydroxybenzophenone with epichlorohydrin at 60 °C for 2 h. Figure S3. UV absorption calibration curves linear fit of (a,b) UV-0 and (c,d) HEPBP. Figure S4. FTIR spectra of neat PBAT and UV-stable PBAT from 850 cm^−1^ to 1350 cm^−1^. Figure S5. XRD patterns of neat PBAT and UV-stable PBAT.
