# Supplementary material for: Design and Preparation of Inherently Photostable Poly(Butylene Adipate-Co-Terephthalate) by Chemically Bonding UV-Stabilizing Moieties in Molecular Chains
Source: Polymers (Basel). 2025 Jun 4;17(11):1567. doi: 10.3390/polym17111567 (PMC12158125; doi:10.3390/polym17111567)
Supplement: Supplementary file 1 [file polymers-17-01567-s001.zip › polymers-3666428-supplementary.pdf]

Supporting information

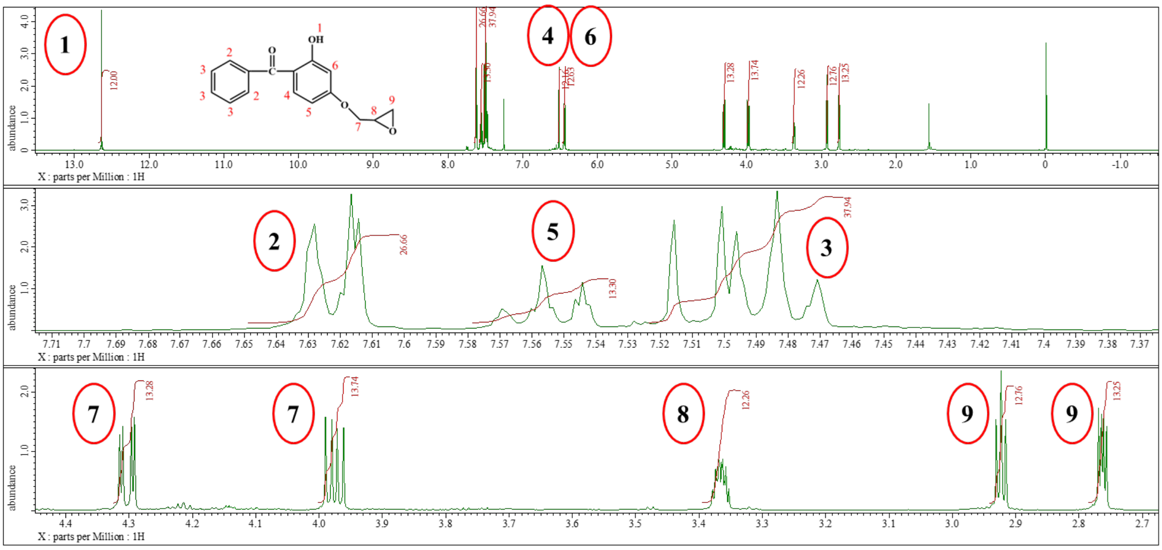

Figure S1. ^{1}H- NMR spectrum of HEPBP.

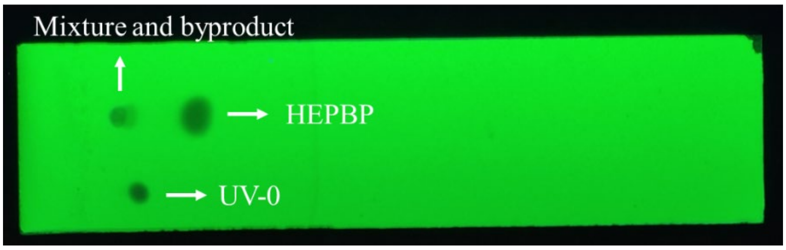

Figure S2. TLC profile of reaction of 2,4-dihydroxybenzophenone with epichlorohydrin at 60 °C for 2h.

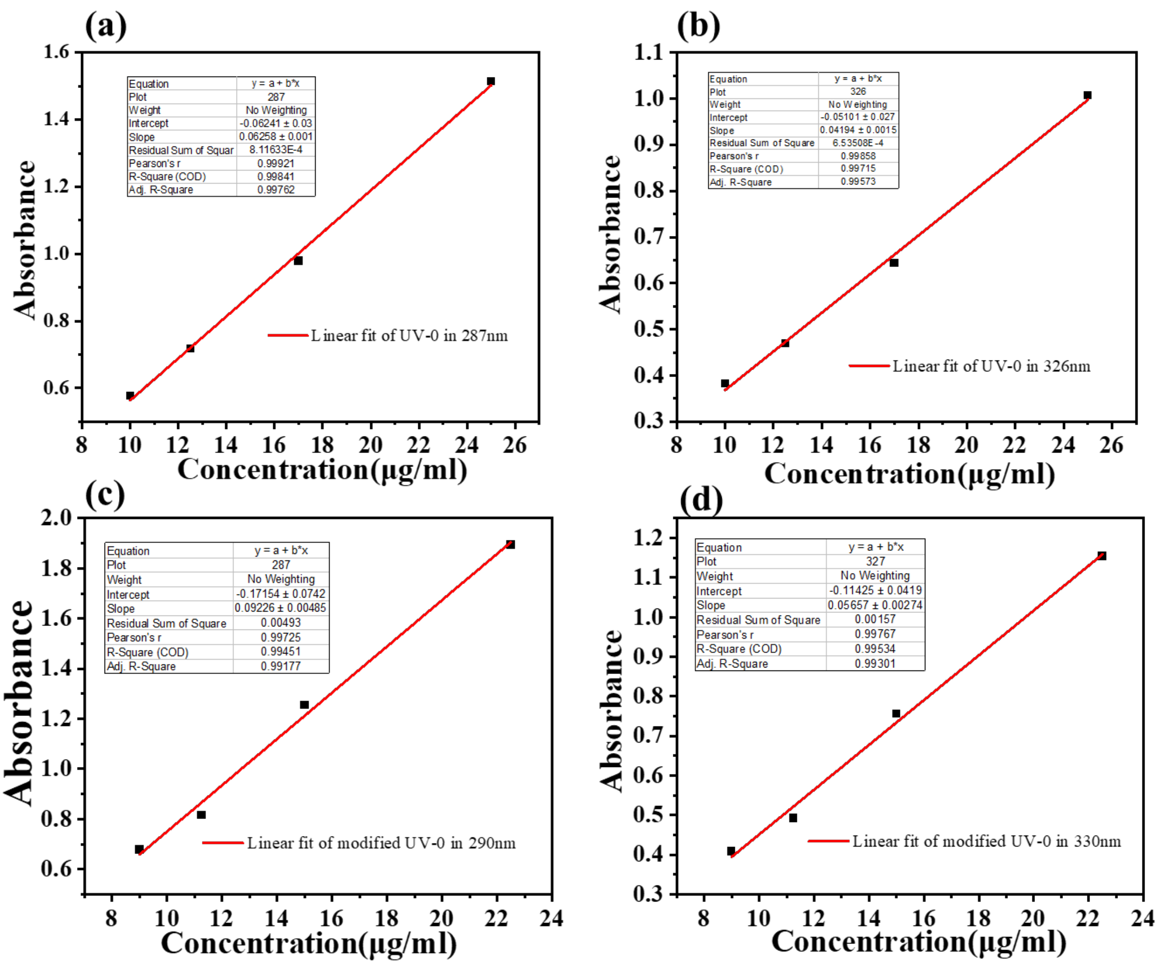

Figure S3. UV absorption calibration curves linear fit of (a,b) UV-0 and (c,d) HEPBP.

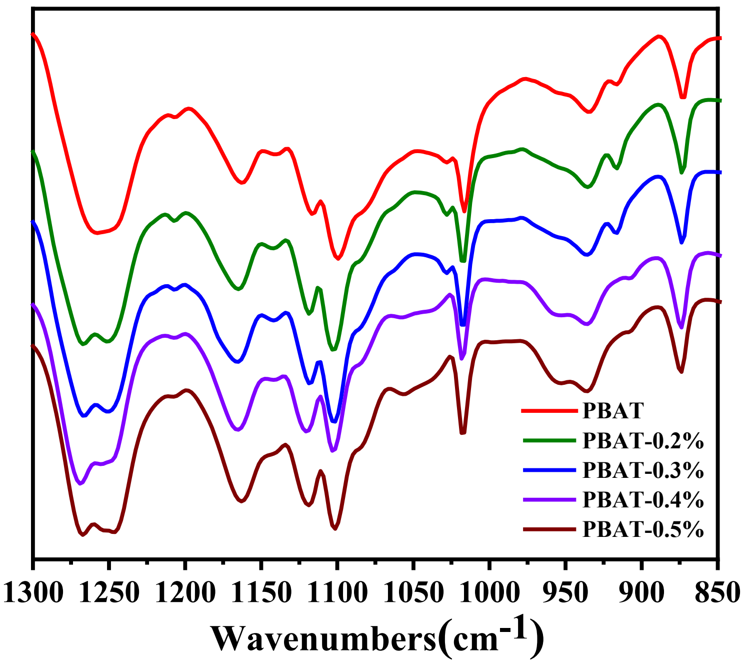

Figure S4. FTIR spectra of neat PBAT and UV-stable PBAT from 850 cm^{-1} to1350 cm^{-1}

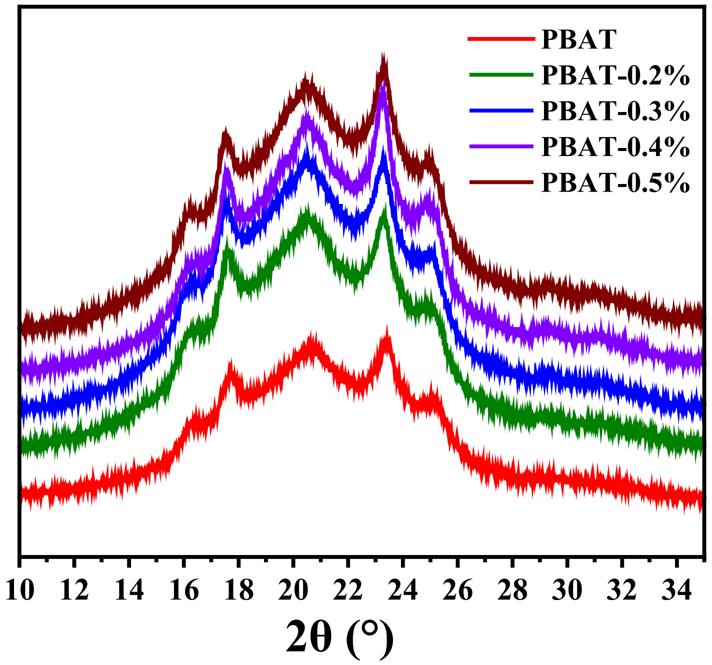

Figure S5. XRD patterns of neat PBAT and UV-stable PBAT.
